# Pleural Mesothelial Cells Modulate the Inflammatory/Profibrotic Response During SARS-CoV-2 Infection

**DOI:** 10.3389/fmolb.2021.752616

**Published:** 2021-11-26

**Authors:** Giulia Matusali, Flavia Trionfetti, Veronica Bordoni, Roberta Nardacci, Laura Falasca, Daniele Colombo, Michela Terri, Claudia Montaldo, Concetta Castilletti, Davide Mariotti, Franca Del Nonno, Maria Rosaria Capobianchi, Chiara Agrati, Marco Tripodi, Raffaele Strippoli

**Affiliations:** ^1^ Laboratory of Virology, National Institute for Infectious Diseases, Lazzaro Spallanzani IRCCS, Rome, Italy; ^2^ Department of Molecular Medicine, Sapienza University of Rome, Rome, Italy; ^3^ Gene Expression Laboratory, National Institute for Infectious Diseases, Lazzaro Spallanzani IRCCS, Rome, Italy; ^4^ Department of Epidemiology, Preclinical Research and Advanced Diagnostics, National Institute for Infectious Diseases “L. Spallanzani” IRCCS, Rome, Italy; ^5^ Laboratory of Electron Microscopy, National Institute for Infectious Diseases “Lazzaro Spallanzani”, IRCCS, Rome, Italy; ^6^ UniCamillus—Saint Camillus International University of Health and Medical Sciences, Rome, Italy

**Keywords:** SARS-CoV-2, mesothelial cells, inflammatory cytokines, pulmonary fibrosis, mesothelial to mesenchymal transition, WT1

## Abstract

Although lung fibrosis has a major impact in COVID-19 disease, its pathogenesis is incompletely understood. In particular, no direct evidence of pleura implication in COVID-19-related fibrotic damage has been reported so far. In this study, the expression of epithelial cytokeratins and Wilms tumor 1 (WT1), specific markers of mesothelial cells (MCs), was analyzed in COVID-19 and unrelated pleura autoptic samples. SARS-CoV-2 replication was analyzed by RT-PCR and confocal microscopy in MeT5A, a pleura MC line. SARS-CoV-2 receptors were analyzed by RT-PCR and western blot. Inflammatory cytokines from the supernatants of SARS-CoV-2-infected MeT5A cells were analysed by Luminex and ELLA assays. Immunohistochemistry of COVID-19 pleura patients highlighted disruption of pleura monolayer and fibrosis of the sub-mesothelial stroma, with the presence of MCs with fibroblastoid morphology in the sub-mesothelial stroma, but no evidence of direct infection *in vivo*. Interestingly, we found evidence of ACE2 expression in MCs from pleura of COVID-19 patients. *In vitro* analysis shown that MeT5A cells expressed ACE2, TMPRSS2, ADAM17 and NRP1, plasma membrane receptors implicated in SARS-CoV-2 cell entry and infectivity. Moreover, MeT5A cells sustained SARS-CoV-2 replication and productive infection. Infected MeT5A cells produced interferons, inflammatory cytokines and metalloproteases. Overall, our data highlight the potential role of pleura MCs as promoters of the fibrotic reaction and regulators of the immune response upon SARS-CoV-2 infection.

## Background

Severe acute respiratory syndrome (SARS)-CoV-2 pathogenesis, characterized by clinical phenotypes spanning from asymptomatic infection to mild disease with symptoms related to airways tract implication, severe pneumonia, acute respiratory distress syndrome (ARDS) and multiple organ failure, remains largely obscure (Hu et al., 2021). Asymptomatic, mild, and severe diseases have been correlated to different cytokine signatures and to differences in innate and adaptive responses to SARS-CoV-2 infection (Bindoli et al., 2020; Carsetti et al., 2020).

It is now well assessed that SARS-CoV-2 first infects epithelial cells of the upper respiratory tract (nasal passages and throat) and especially lungs (bronchi and alveoli), where alveolar type I and type II cells (AT1 and AT2, respectively) are believed to mediate the first encounter with the virus; infection of alveolar macrophages is determinant in mediating the amplification of the inflammatory and immune responses (Grant et al., 2021).

Mechanistically, cell entry is mediated by the engagement of the receptor angiotensin-converting enzyme (ACE2) (Grant et al., 2021). ACE2 is also expressed by cells in the kidney, blood vessels, heart, whose infection by SARS-CoV-2 may mediate the characteristic multi-organ pathology (Grant et al., 2021). Viral uptake is also promoted by transmembrane protease serine (TMPRSS)2, disintegrin and metallopeptidase domain (ADAM)17 cleaving ACE2, in addition to activating the S protein of the virus for membrane fusion (Heurich et al., 2014; Hoffmann et al., 2020). Neuropilin-1 (NRP1) also potentiates SARS-CoV-2 cell entry and infectivity (Cantuti-Castelvetri et al., 2020).

Similarly to the other serosal membranes (i.e., peritoneum, and pericardium), pleura is lined by a monolayer of mesothelial cells (MCs) arranged on a basement membrane, which separates it from the submesothelial stroma (Mutsaers et al., 2015). MCs differentiation is controlled by the transcription factor Wilms’ tumor (WT)1, which is commonly used for lineage-tracing experiments (Gulyas and Hjerpe, 2003; Karki et al., 2014). The main function of MCs resides in the creation of a slippery non-adhesive surface allowing frictionless movements between adjacent parietal and visceral surfaces (Mutsaers et al., 2015). Moreover, MCs favor leukocyte recirculation and regulate the development of immune responses (Terri et al., 2021).

With respect to pathological conditions such as infections, MCs are a key factor in driving the immune response through the production of large quantities of extracellular mediators such as inflammatory cytokines and chemokines (Terri et al., 2021). Moreover, inflammatory and infectious stimuli may promote a complex multistep phenomenon called mesothelial to mesenchymal transition (MMT) (Lopez-Cabrera, 2014). In this context, MCs progressively lose epithelial-like features, acquiring new mesenchymal-like invasive and profibrotic abilities. Lineage-tracing of WT1-positive MCs in a context of fibrotic lung disease provided evidence of MMT induction *in vivo* (Sontake et al., 2018).

Due to its anatomic localization, pleura may be affected by many viral infections of the respiratory tract including influenza, coxsackievirus, respiratory syncytial virus (RSV), cytomegalovirus (CMV) (Nestor et al., 2013).

In spite of the conceivable MCs participation in COVID-19 pathogenesis, so far only indirect evidence has been reported. In this study, we highlight that SARS-CoV-2 causes structural modifications in the pleura with disruption of the mesothelial monolayer and the generation of WT1/cytokeratin-positive cells infiltrating the sub-mesothelial stroma. When analysing cellular/molecular mechanisms underlying this event, we found that MeT5A cells (a pleura non tumorigenic mesothelial cell line) express specific receptors and coreceptors for SARS-CoV-2 and produce infectious viral particles. Moreover, MeT5A cells infection resulted in the production of a broad repertoire of interferons, pro- and anti-inflammatory cytokines, and metalloproteases (MMPs).

Overall, this study provides a first evidence on a specific involvement of pleura MCs in SARS-CoV-2 pathology.

## Methods

### Reagents and Antibodies

Polyclonal antibody against WT1 (#12609-2-AP) was from Proteintech (Rosemont, IL); anti SARS-CoV Nucleocapsid (#200-401-A50) for confocal microscopy experiments was from Rockland Immunochemicals, Inc. (Limerick, PA. United States); anti SARS-CoV Nucleoprotein/NP for immunohistochemistry experiments was from Sino Biological, (40143-T62, Beijing, China); anti ACE2 (AB_2792286) was from Invitrogen (Waltham, MA United States); anti ADAM17 (AB_10980438) was from Invitrogen. Monoclonal antibody anti dsRNA (10010200) was from Nordic-MUbio (Rangeerwe, Netherlands), anti-cytokeratin AE1/AE3/PCK26 (760-2595) was from Ventana (Oro Valley, Arizona. United States); anti-hsp90 (sc-13119) was from Santa Cruz Biotechnology (Dallas, TX United States); anti activated caspase 3 (9661) was from Cell Signaling technology (Danvers, MA, United States); anti TMPRSS2 (H-4: sc-515727) was from Santa Cruz Biotechnology; anti-GAPDH (cb1001) was from Calbiochem (Kenilworth, NJ, United States). DRAQ5 staining solution (#130-117-343) was from Miltenyi Biotec (Bergisch Gladbach, Germany). Sodium arsenite was from Sigma-Aldrich (Saint Louis, MO United States).

### Cells

The human mesothelial cell line MeT5A (ATCC, Rockville, MD) was cultured in Earle’s M199 supplemented with 10% fetal calf serum, 50 U/ml penicillin, 50 μg/ml streptomycin (Sigma-Aldrich).

Vero E6 cells (ATCC) were cultured in Eagle’s Minimum Essential Medium supplemented with 10% fetal calf serum, 50 U/ml penicillin, 50 μg/ml streptomycin.

### Viral Infection

Subconfluent MeT5A (200.000 cells/well) were incubated with SARS-CoV-2 (SARS-CoV-2 isolate SARS-CoV-2/Huma n/ITA/PAVIA1073 4/2020, clade G, D614G (S) obtained from Dr. Fausto Baldanti, Policlinico San Matteo, Pavia, Italy) in serum-free Eagle’s Minimum Essential Medium at a multiplicity of infection (M.O.I) of 1 for 1.5 h at 37°C, 5% CO_2_. Then, cells were washed three times with PBS to remove viral inoculum, and complete culture medium was added. Culture supernatants and cell lysates were collected at 1.5, 24, and 72 h post infection (p.i.). Statistical significance was determined with a nonparametric Wilcoxon signed rank test with GraphPad Prism version 8.0 (La Jolla, CA, United States). Differences were considered significant at *p* < 0.05.

### RT-PCR

Viral RNA was extracted from 140 μl of culture supernatant using the Qiamp viral RNA kit (Qiagen, Hilden, Germany), following manufacturers instruction and eluted in 50 μl of elution buffer.

Real time RT-PCR to analyze viral genome was performed on 10 μl of RNA extracted from cell culture supernatant, or 40 ng of cell-associated RNA using the RealStar^®^ SARS-CoV-2 RT-PCR Kit RUO (Altona Diagnostics, Hamburg, Germany), which amplifies the E- and S- viral genes.

Cellular RNA was extracted from cell cultures using TRIzol reagent (Life Technologies, Carlsbad, CA), according to the manufacturer’s instructions. cDNA synthesis was generated using a reverse transcription kit (A3500) from Promega (Madison, WI), according to the manufacturer’s recommendations.

cDNAs were amplified by qPCR reaction using Maxima SYBR Green/ROX qPCR Master Mix (K0253) from Thermo Fisher Scientific (Waltham, MA). qPCR reactions were performed with the Rotor-Gene 6000 thermocycler (Corbett Research, Cambridge, United Kingdom). The primer sequences used in this study are shown in Table 1.

**TABLE 1:** List of RT-PCR primers used in this study.

Gene	Forward sequence	Reverse sequence
hACE2	GGG​ATC​AGA​GAT​CGG​AAG​AAG​AAA	AGG​AGG​TCT​GAA​CAT​CAT​CAG​TG
hADAM17	GGG​CAG​AGG​GGA​AGA​GAG​TA	GAC​TTG​AGA​ATG​CGA​ATC​TGC​T
hIFNα	TGG​TGC​TCA​GCT​ACA​AAT​CC	CCC​ATT​TGT​GCC​AGG​AGT​AT
hIFNβ	TGG​GAG​GCT​TGA​ATA​CTG​CCT​CAA	TCT​CAT​AGA​TGG​TCA​ATG​CGG​CGT
hL34	GTC​CCG​AAC​CCC​TGG​TAA​TAG​A	GGC​CCT​GCT​GAC​ATG​TTT​CTT
hNRP1	AAG​GTT​TCT​CAG​CAA​ACT​ACA​GTG	GGG​AAG​AAG​CTG​TGA​TCT​GGT​C
hTMPRSS2	AAT​CGG​TGT​GTT​CGC​CTC​TAC	CGT​AGT​TCT​CGT​TCC​AGT​CGT

Relative amounts, obtained with 2 (−ΔCt) method, were normalized with respect to the housekeeping gene L34. Statistical significance was determined with a *t* test with Prism version 8.0. Differences were considered significant at *p* < 0.05. Values are reported in the graphs.

### Western Blotting

MeT5A cells were lysed in CelLytic™ MT Cell Lysis Reagent supplemented with 1 mM PMSF; 1 μg/ml each of aprotinin, leupeptin and pepstatin; and 25 mM NaF (all from Sigma). Equal amounts of protein were resolved by SDS-PAGE. Proteins were transferred to nitrocellulose membranes (Amersham Life Sciences, Little Chalfont, United Kingdom) and probed with primary antibodies using standard procedures. Peroxidase–conjugated secondary antibodies anti-rabbit (711-036-152) and anti-mouse (715-036-150) were from Jackson Immuno Research Laboratories, (West Grove, PA, United States). Nitrocellulose bound antibodies were detected by enhanced chemiluminesence (ECL) Immobilon Classico WBLUC0500 and Immobilon Crescendo Western HRP substrate WBLUR0500 from Millipore (Burlington, MA, United States).

### Viral Titration

To estimate the production of infectious SARS-CoV-2, serial dilutions of MeT5A cell culture supernatants were put in contact with sub-confluent Vero E6 cells seeded in 96-well plates. At day 5 after infection, cells were observed for Cytopathic effect (CPE) and tissue culture infective dose (TCID) 50/ml was measured and analyzed by Reed-Muench method.

### Confocal Microscopy

72 h after infection, SARS-CoV-2 infected and not infected cells were washed in cold PBS, fixed with 4% paraformaldehyde (Sigma-Aldrich) in PBS and permeabilized with 0.2% Triton X-100 (Sigma-Aldrich) in PBS. Alexa Fluor 488 secondary antibody was from Thermo Fisher Scientific; Cy3-conjugated secondary antibody was from Jackson Immunoresearch (Philadelphia, PA). Coverslips were mounted in Prolong Gold antifade (Life Technologies) and examined under a confocal microscope (Leica TCS SP2, Wetzlar, Germany). Digital images were acquired with the Leica software and the image adjustments and merging were performed by using the appropriated tools of ImageJ software. A minimum of 4 fields per sample (at least 150 total cells per total) from two independent experiments was analyzed.

### Viability Assay

Cell viability was evaluated by ViaKrome 808 Fixable Viability Dye (Beckman Coulter) according to manufacturer’s instructions. Cells were stained at 24 and 72 h after infection, fixed with 1% Paraformaldehyde (PFA) (Bio-Rad laboratories, Hercules, CA, United States) and washed 1x PBS. Data were recorded with a Cytoflex LX cytometer running CytoExpert Software (Beckman Coulter). Three independent experiments were performed. n.s.: not significant.

### Cytokine Detection

Supernatants from SARS-CoV-2 infected MeT5A cell cultures were collected at 24 and 72 h after infection. We performed multianalyte profiling of 37 cytokines, chemokines, and soluble mediators in the supernatants of all samples, using the Luminex based multiplex bead technology (Bio-Plex Pro Human Cytokine Panel group I: APRIL/TNFSF13, BAFF/TNFSF13B, sCD30/TNFRSF8, sCD163, Chitinase-3-like 1, gp130/sIL-6Rβ, IFN-α2, IFN-β, IFN-γ, IL-2, sIL-6Rα, IL-8, IL-10, IL-11, IL-12 (p40), IL-12 (p70), IL-19, IL-20, IL-22, IL-26, IL-27 (p28), IL-28A/IFN-λ2, IL-29/IFN-λ1, IL-32, IL-34, IL-35, LIGHT/TNFSF14, MMP-1, MMP-2, MMP-3, Osteocalcin, Osteopontin, Pentraxin-3 sTNF-R1 sTNF-R2, TSLP, TWEAK/TNFSF12, Biorad). The assay was conducted accordingly to manufacturer’s recommendations. Plates were measured using the Bio-Plex MagPix System and analyzed with the Bio-Plex Manager version 6.0 (BioRad Laboratories).

IL1-β, IL-6, IL-8 and TNF-α were measured in supernatants samples by using an automated ELISA assay (ELLA microfluidic analyzer, Protein Simple, San Jose, CA, United States).

Statistical significance was determined with a *t*-test with GraphPad Prism version 8.0. Differences were considered significant at *p* < 0.05.

### Autoptic Lung and Pleura

Lung tissue samples, including pleura, were obtained from post-mortem examination of four SARS-CoV-2-infected patients, performed at the National Institute for Infectious Diseases Lazzaro Spallanzani-IRCCS Hospital (Rome, Italy). All patients were diagnosed as COVID-19 by SARS-CoV-2 RT-PCR performed on nasopharyngeal and oropharyngeal swabs. Demographics and clinical course of patients are shown in Table 2.

**TABLE 2:** Demographic and clinical features of COVID-19 patients.

Patient number	Gender	Age	Comorbidities	Causes of death
1	M	81	Hypertension cardiomyopathy Aortic aneurysm	Myocardial infarction. Diffuse alveolar damage (ARDS). Interstitial pneumonia
2	M	54	None	Interstitial pneumonia. Myocarditis
3	M	82	Not known	Interstitial pneumonia. Cardiorespiratory failure
4	M	74	Hypertension. Knee arthroplasty	Interstitial pneumonia. Cardiorespiratory failure

Autopsies were performed according to guidance 167 for post-mortem collection and submission of specimens and biosafety practices to reduce the risk of transmission of infectious pathogens during and after the post-mortem examination (Hanley et al., 2020). Lungs samples, including pleura, of four non-COVID-19 patients were used as comparative controls (Table 3).

**TABLE 3:** Demographic and clinical features of non-COVID-19 patients.

Patient number	Gender	Age	Comorbidities	Causes of death
1	M	58	Hemicolectomy	H1N1 Pneumonia
2	M	43	Alcoholic cirrhosis	Interstitial pneumonia and pulmonary fibrosis
3	M	47	None	Cardiorespiratory failure
4	F	47	Surgery for frontotemporal meningioma and kidney cancer	Myocarditis and Interstitial pneumonia

The study was approved by the local Clinical Research Ethics Committee (approval number: no 9/2020). Written informed consent was waived by the Ethics Commission for both COVID-19 and non COVID-19 patients due to public health outbreak investigation. Specimens from lung tissues were fixed in 10% neutral buffered formalin, and routinely processed to paraffin blocks.

### Immunohistochemistry of Pleura

Deparaffinized and rehydrated sections were used for immunohistochemistry. Organ sections were immersed in 10 mM sodium citrate, pH 6.0 and microwaved for antigen retrieval and immunostained on BenchMark ULTRA system fully automated instrument (Roche, Basel, Switzerland). All cases were independently analyzed by two pathologists.

## Results

### Myofibroblast Transformation of MCs in Visceral Pleura From SARS-CoV-2-Infected Patients

We previously demonstrated that SARS-CoV-2 infection caused a multisystem pathology with a dominant pulmonary and cardiovascular involvement (Falasca et al., 2020). In the present study, autoptic visceral pleura from COVID-19 patients was analyzed and compared to visceral pleura from non-COVID-19 patients.

**FIGURE 1:**
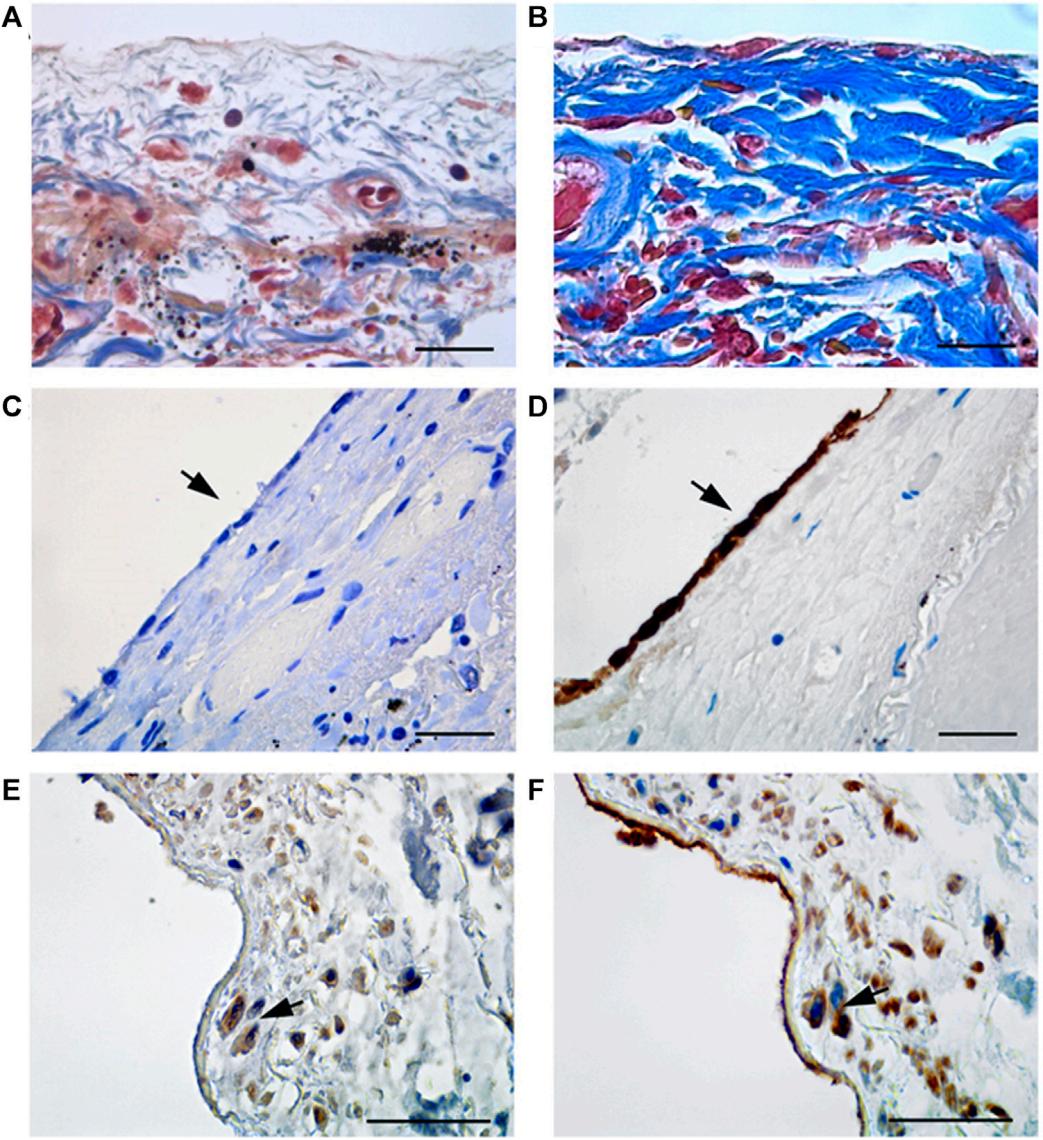
**(A)** Masson’s trichrome staining in tissue from non COVID-19 patients; **(B)** Masson’s trichrome staining highlights the presence of collagen fibers (blue stain) in thickened submesothelial layer of visceral pleura from COVID-19 patients. **(C)** Visceral pleura from non COVID-19 patients show absence of staining (arrow) for WT1, a marker of reactive mesothelial cells. **(D)** Keratin AE1/AE3 staining, marker of mesothelial cells, shows a continuous monolayer of MCs in non COVID-19 patients. **(E,F)** Immunohistochemical labeling of WT1 shows positive submesothelial spindle cells in pleura from COVID-19 patients (E, arrow), and Keratin AE1/AE3 staining, performed on a consecutive section, show that the same cells (F, arrows) express both markers. Scale bars = 30 µm.

Masson’s trichrome staining revealed the onset of an intense fibrotic response in samples from COVID-19 patients (Figure 1B), with respect with non COVID-19 patients (Figure 1A). When analyzing pleura cellular components, while the MCs monolayer was maintained in pleura non-COVID-19 patients (Figures 1C,D), it appeared almost totally lost COVID-19 patients (Figures 1E,F), highlighting the specificity of pleural disruption in this disease.

Immunohistochemical labeling with WT1, a MC marker, showed positivity in spindle-like cells infiltrating the sub-mesothelial stroma of COVID-19 patients (Figure 1E). Accordingly, staining with anti-cytokeratin AE1/AE3 antibody confirmed the mesothelial origin of these infiltrating cells (Figure 1F). The sub-mesothelial stroma in non-COVID-19 samples was devoid of WT1, (Figure 1C), or cytokeratin positive cells, (Figure 1D), highlighting a specific impact of SARS-CoV-2 infection in promoting the acquisition of invasive ability by MCs. Anti-SARS-CoV immunolabeling did not reveal specific stain in the rare MCs present in visceral pleura from COVID-19 patients (Supplementary Figure S1A). However, positivity was found in pneumocytes from the same patients (Supplementary Figure S1B), in agreement to previously published data (Falasca et al., 2020).

These results demonstrate that SARS-CoV-2 infection causes the disruption of the monolayer of epithelial-like MCs, which in turn may invade the sub-mesothelial stroma promoting the onset of pleural fibrosis.

### MCs Support SARS-CoV-2 Infection/Replication

To characterize cellular and molecular mechanism underlying the observed lung alterations *in vivo*, we made use of MeT5A, a pleura non-transformed MC line widely used in the study of pleura pathophysiological functions, such as mesothelial plasticity and fibrosis (Strippoli et al., 2008; Murphy et al., 2012; Rossi et al., 2018; Woo et al., 2021).

**FIGURE 2:**
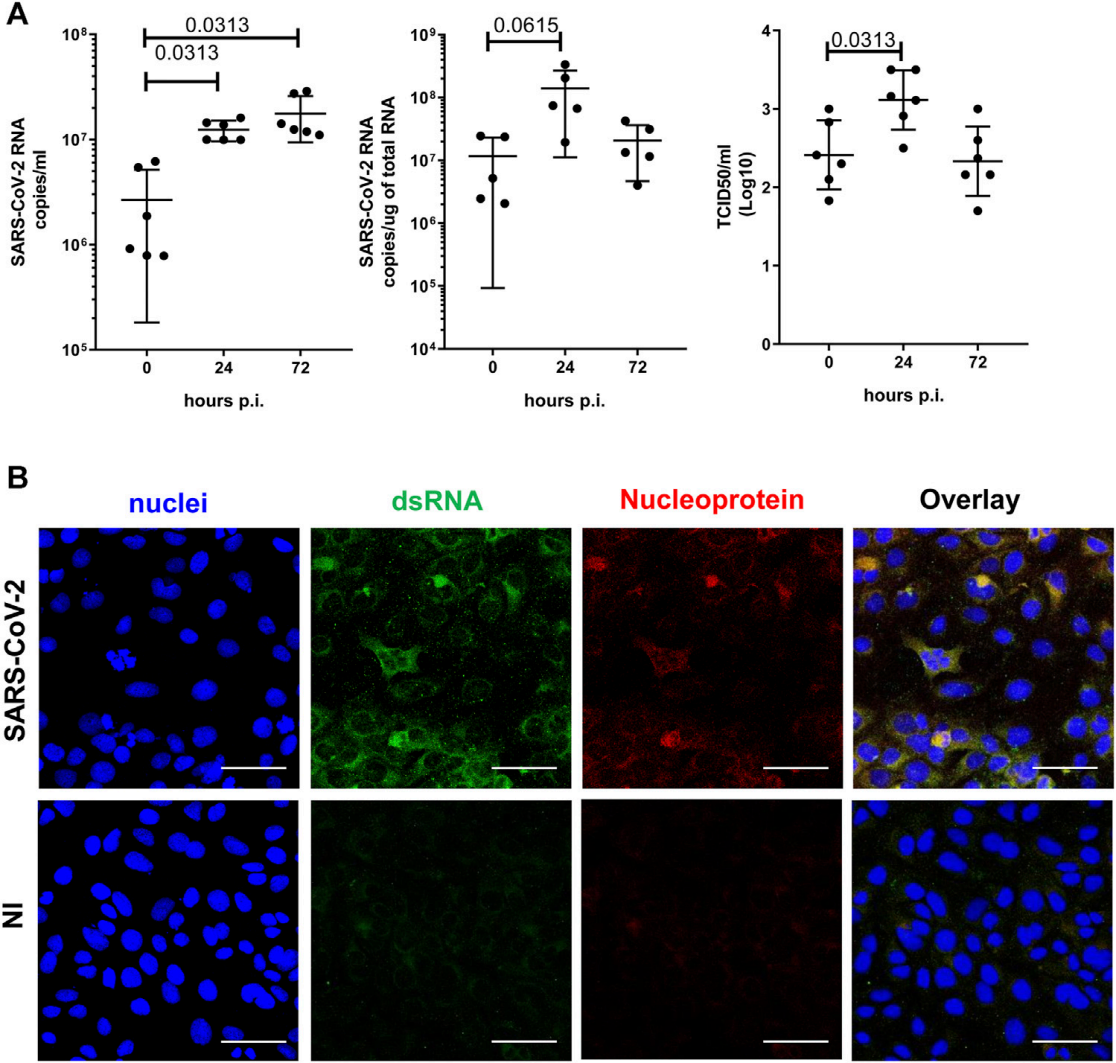
**(A)** Left: Quantification of SARS-CoV-2 viral RNA expression in culture supernatants of MeT5A cells at 1.5, 24 and 72 h post viral inoculum (M.O.I. of 1). Six independent experiments were performed. Middle: Quantification of SARS-CoV-2 viral RNA expression in total RNA of MeT5A cultured as above. Five independent experiments were performed. Right: A TCID50 (Median Tissue Culture Infectious Dose) assay was performed adding serial dilutions of MeT5A cell culture supernatants to sub-confluent VeroE6 cells seeded in 96-well plates. Six independent experiments were performed. *P* was calculated with respect to time 0 of infection. Differences were considered significant at *p* < 0.05. **(B)** Immunofluorescence of MeT5A cells exposed for 120 h to SARS-CoV-2 (M.O.I. of 1) compared with non-infected (NI) cells. Fixed cells were stained with antibodies against SARS-CoV Nucleocapsid and dsRNA. Nuclei were stained with DRAQ5. A minimum of 150 cells per sample from two independent experiments were analyzed. Scale bar: 50 μm.

SARS-CoV-2 infection (M.O.I. of 1) of MeT5A resulted in a progressive accumulation of viral RNA in the supernatants at 24 (fold increase mean 8.2) and 72 (fold increase mean 16.4) hours p.i. (Figure 2A, left). Moreover, intracellular SARS-CoV-2 RNA peaked at 24 and slightly declined at 72 h p.i (Figure 2A middle). The presence of infectious SARS-CoV-2 viral particles in MeT5A supernatants was demonstrated by productive infection of Vero E6 cells (Figure 2A right). To further confirm viral infection of MeT5A, double-strand (ds) RNA and viral nucleoprotein (N) were detected by confocal microscopy at 72 h p.i. (Figure 2B). Exposure of MeT5A cells to SARS-CoV-2 viral particles did not cause an evident cytopathic effect and cell death, as demonstrated by bright-field microscopic analysis (not shown), by a ViaKrome viability assay and by cleaved caspase 3 detection (Supplementary Figures S2A,B).

**FIGURE 3:**
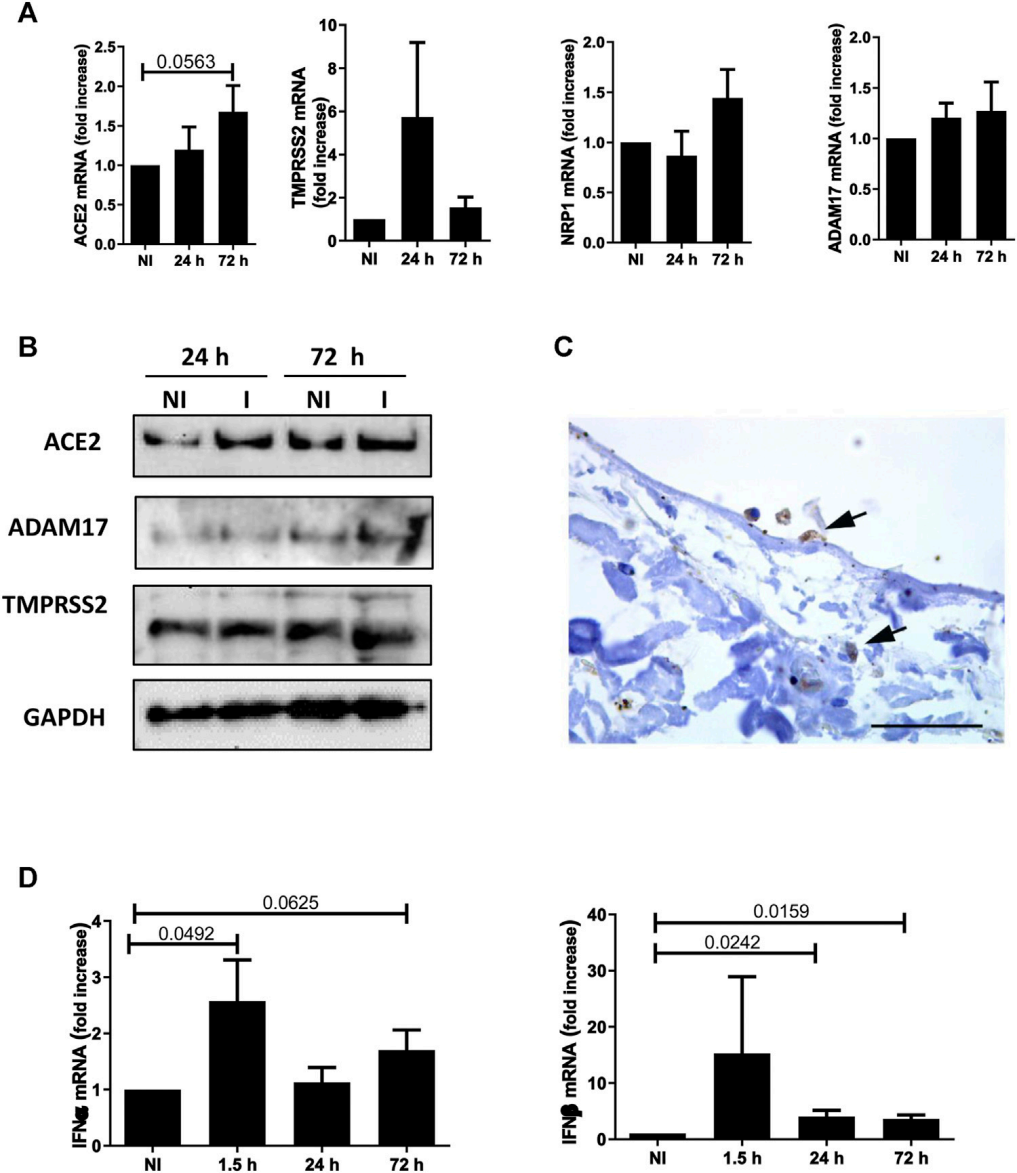
**(A)** Quantitative RT-PCR expression analysis of *ACE2*, *TMPRSS2*, *NRP1* and *ADAM17* from total RNA of MeT5A cells exposed to SARS-CoV-2 infection (MOI = 1) for 24 or 72 h compared with non-infected (NI) cells. L34 mRNA levels were used for normalization. Bars represent the mean ± SEM of triplicate determinations in at least four independent experiments. *P* was calculated with respect to NI samples. Differences were considered significant at *p* < 0.05. **(B)** Western blot showing the expression of ACE2, TMPRSS2 and ADAM17, SARS-CoV-2 plasma membrane receptors, from total lysates of MeT5A cells treated as in A. I: SARS-CoV-2 infected cells. GAPDH was detected as a loading control. **(C)** Labeling with a specific antibody provides evidence of ACE2 expression (arrows) in MCs from visceral pleura of COVID-19 patients. Scale bar: 30 μm. **(D)** Quantitative RT-PCR expression analysis of *IFNα* and *IFNβ* in MeT5A cells exposed to SARS-CoV-2 for 1.5, 24 or 72 h (M.O.I. of 1) compared with NI cells. Quantitative RT-PCR was performed on total RNA. L34 mRNA levels were used for normalization. Bars represent the mean ± SEM of triplicate determinations in at least four independent experiments. *P* was calculated with respect to NI samples. Differences were considered significant at *p* < 0.05.

To provide mechanistic evidence on SARS-CoV-2/MC interactions, we analyzed the expression of the plasma membrane receptors implicated in viral entry, namely ACE2, the protease TMPRSS2, and the co-factors NRP1 and ADAM17. As shown in Figure 3A, MeT5A cells express ACE2, TMPRSS2, NRP1, and ADAM17. As demonstrated by kinetic infection studies, ACE2 expression has a trend to increase after SARS-CoV-2 infection, whereas no changes in expression of the other receptors were observed. Expression of ACE2, TMPRSS2 and ADAM17 was confirmed at protein level by western blot analysis (Figure 3B). Interestingly, we found evidence of ACE2 expression (the main SARS-CoV2 plasma membrane receptor) in pleura MC by IHC (Figure 3C).

With respect to cellular specific response, SARS-CoV-2 infection promoted a rapid induction of Type I interferons (IFN-I), as demonstrated by IFN-α and -β mRNA expression already induced at 1.5 and still significantly expressed at 72 h upon infection (Figure 3D).

### The Infection of MCs by SARS-CoV-2 Promotes Cytokine Production

**FIGURE 4:**
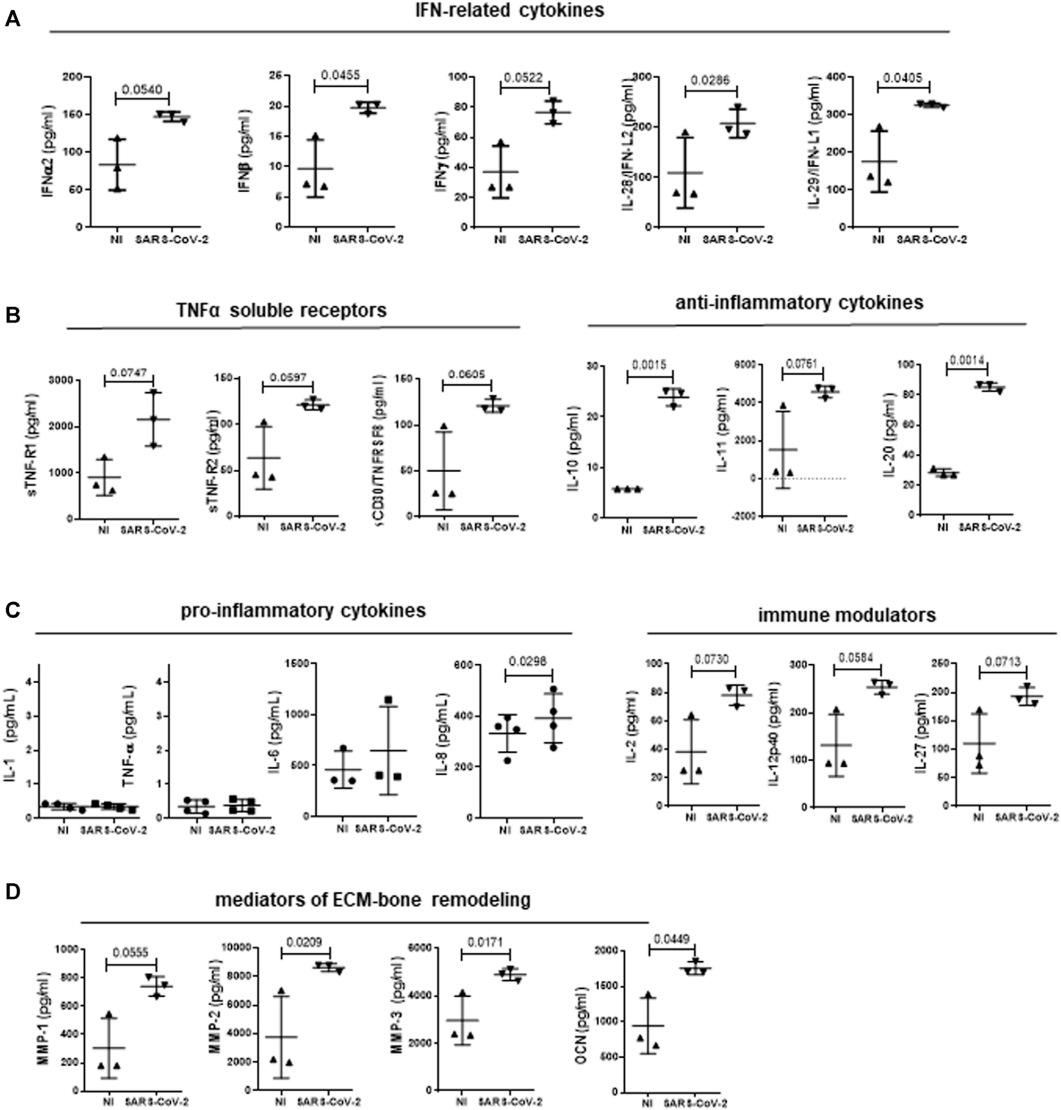
Analysis of cytokine secretion from supernatants of MeT5A cells infected with SARS-CoV-2 or left uninfected for 72 h (M.O.I. of 1). SARS-CoV-2 infection promoted the production of IFN-related cytokines **(A)**; TNFα-soluble receptors **(B, left)**; anti-inflammatory cytokines **(B, right)**; pro-inflammatory cytokines **(C, left)**; immune modulators **(C, right)**; mediators of ECM-bone remodeling **(D)**. *P* was calculated with respect to NI samples. Differences were considered significant at *p* < 0.05.

The specific contribution of the infected MCs in the modulation of the inflammatory response and extracellular matrix (ECM) remodeling was therefore explored. Supernatants from SARS-CoV-2-infected MeT5A cells were analyzed at 24 and 72 h after infection. 37 extracellular inflammatory mediators were evaluated by Luminex technology. Furthermore, the analysis was extended to another panel of inflammatory cytokines (IL-1β, TNFα, IL-6, IL-8) measured by automatic ELLA assay. The complete list and values of cytokines analyzed, including those not significantly expressed/modulated is shown in Supplementary Figure S3, whereas significant induction of cytokines (observed at 72 h after infection) in shown in Figure 4.

The induction of an IFN response previously observed at mRNA level was confirmed by the presence of increased levels of IFNα−β−γ and -λ (IL-28-29) (Figure 4A). Secretion of cytokines with inhibitory activity belonging to TNF superfamily, (sTNF-R1, sTNF-R2 and sCD30/TNFRSF8) was increased (Figure 4B, left). On the other hand, production of TNFα and IL-1β, that are known to be secreted by MCs, was negligible upon SARS-CoV-2 infection (Douvdevani et al., 1994; Raby et al., 2018) (Figure 4C, left). Interestingly, the increase in the production of IL-10 and the structurally related IL-20 was highly significant (Figure 4B, right), whereas the abundant production of IL-6, characteristic of these cells, was not significantly increased by SARS-CoV-2 infection (Figure 4C, left) (Fujino et al., 1996). Of note, IL-8 production was significantly increased upon SARS-CoV-2 infection (Figure 4C, left). Moreover, MCs produced increased amounts of IL-2, IL-12p40 and IL-27, known modulators of innate and adaptive immunity (Figure 4C, right). Last, MCs secreted increased levels of MMP1-3, and of osteocalcin (Figure 4D). These data suggest that infected MCs may impact both the immune response to SARS-CoV-2 via the predominant production of anti-inflammatory mediators and the modification of the pleural stroma via the production of ECM remodelers.

## Discussion

Our observations provide a first evidence of SARS-CoV-2-infection/replication and inflammatory cytokine secretion by pleura MCs. Moreover, immunohistochemical analysis of samples from COVID-19 patients demonstrated a specific alteration of pleura characterized by disruption of the MC monolayer and invasion of the sub-mesothelial stroma by spindle-like MCs.

These data fall in a context where pleura-specific role in SARS-CoV-2 pathology has not yet been clarified. In particular, no evidence so far pointed to a direct role of pleura MCs in the COVID-19 pathogenesis. This in spite of the fact that around 10% of patients develop pleural effusions, have higher incidence of severe/critical illness, mortality rate, and longer hospital stay time compared to their counterparts without pleural effusion (Luo et al., 2020; Mo et al., 2020; Zhan et al., 2021). While there are reports linking fibrosis onset with disease severity, the cellular and molecular mechanisms have been incompletely studied so far.

Indeed, circulating ECM components have been correlated with prognosis (Ding et al., 2020). In COVID-19 patients, radiographic evidence of a wide range of pulmonary alterations associated to fibrotic damage and lung functional impairment are commonly observed (Shi et al., 2020). In fatal cases, increased expression of ECM-specific proteins and ECM regulators has been reported (Skibba et al., 2020). These data suggest that lung fibrosis onset is a pathogenic mechanism of severe SARS-CoV-2 infections. Accordingly, post infectious pulmonary fibrosis is also a known outcome in survivors of SARS, an infection with the closely related virus, SARS-CoV (Hui et al., 2005).

In this study, the observation of MCs loss with disruption of the MCs monolayer in biopsies of visceral pleura from autopsies of COVID-19 patients leads to hypothesize a direct or cell-mediated cytopathic effect of the virus. In particular, WT1-and cytokeratin-positive cells with a fibroblastoid morphology (having undergone bona fide MMT) were found the sub-mesothelial stroma.

Since MCs form in normal conditions a continuous monolayer above the basal membrane, the observation of MCs in the sub-mesothelial stroma during SARS-CoV-2 infection implicates the onset of an invasive program in these cells. However, the limited number of patients analyzed in this study made impossible to make correlations with clinical data and to further characterize the underlying invasive process.

Events linked with MCs plasticity may influence the fibrotic process in different ways. MMT in particular, has been demonstrated as a common mechanism of fibrosis in serosal membranes exposed to biomechanical, inflammatory and infectious stimuli (Ruiz-Carpio et al., 2017; Namvar et al., 2018; Raby et al., 2018; Strippoli et al., 2020).

Once transdifferentiated, MCs produce abundant amounts of TGFβ1, fibronectin, collagens (Mutsaers et al., 2015; Rossi et al., 2018). These cells may also rearrange the ECM through the expression of contractile proteins (i.e., αSMA) as well as the production of MMPs, such as MMP-2, -9 and -14 or MMP inhibitors such as TIMP1 and PAI1 (Ma et al., 1999; Strippoli et al., 2020).

So far, only indirect evidence pointed to an ability of SARS-CoV-2 to infect MCs. Viral load has been occasionally demonstrated in pleural fluid of infected patients; in this case, pleura MCs were found with large multiple nuclei, consistent with a cytopathic effect of the virus, although an infection of MCs by SARS-CoV-2 was not been formally proved (Bennett et al., 2020; Malik et al., 2020; Mei et al., 2020). Furthermore, an indirect evidence of a role of MCs is the fact that pleural effusions are linked with the onset of pleural fibrosis, which, along with pericarditis, is frequently found during COVID-19 (Falasca et al., 2020; Zeng et al., 2020). Indeed, MCs are key determinant of lung fibrotic diseases (Mutsaers et al., 2015; Liu et al., 2020).

In this study, we demonstrated that MeT5A cells express the main entry factors implicated in SARS-CoV-2 infection, i.e. ACE2, TMPRRS2, ADAM17 and NRP1. Cleavage of ACE2 has been demonstrated to impact on viral entry (Hoffmann et al., 2020). While it is already known that MCs express high levels of NRP1, a coreceptor of VEGFR with pro-fibrotic activity, the expression of ACE2, TMPRSS2 and ADAM17 has not been reported so far (Perez-Lozano et al., 2013; Cantuti-Castelvetri et al., 2020).

*ACE2* levels in airway epithelial cells have been demonstrated to increase during SARS-CoV-2 infection, potentially rendering these patients even more vulnerable to SARS-CoV-2 (Chua et al., 2020). Other events, such as cigarette smoke may also increase ACE2 levels provoking SARS-CoV-2 increased infectivity in these individuals (Liu et al., 2021).

Our data suggest that ACE2 is already expressed in MCs in basal conditions, and the modulation of its expression is not a major mechanism regulating SARS-CoV-2 infection in these cells; in fact we found an high expression of ACE2 in uninfected MeT5A cells, and a tendential increase upon infection with SARS-CoV-2.

SARS-CoV-2 has a strong specificity in terms of cellular effects. For instance, SARS-CoV-2 promotes apoptosis in VERO3, but not in CALU3 cells (Park et al., 2021). In these cell lines, the induction of apoptosis has been related to high virus production and limited induction of interferon response. Moreover, cell culture conditions are extremely relevant to study SARS-CoV-2 effects in the lung and other organs. For instance, freshly isolated primary alveolar cultures from healthy individuals were found only minimally susceptible to SARS-CoV-2 (Hou et al., 2020).

Our data suggest that apoptosis is not induced in infected MeT5A cells. It seems conceivable that in our experimental conditions MeT5A survival is linked to the induction of Interferon response, which provokes the progressive clearance of infection over time. Apoptosis observed *in vitro* in other cell types (e.g., AT2 cells) may be influenced by specific experimental systems used, such as the use of feeder layer or organoids (Mulay et al., 2021; Katsura et al., 2020; Van Der Vaart et al., 2021). In these latter soft ECM-3D cultures often used to prevent AT2 cell differentiation, the induction of apoptosis may be relatively favored (Zhang et al., 2011; Halder et al., 2012).

By means of multiple approaches, we demonstrated that MCs sustain SARS-CoV-2 infection. Moreover, with respect to the cellular response to infection, we found an early (1.5 h post inoculum) induction of IFN-α and -β mRNA upon treatment with SARS-CoV-2. While the induction of a rapid IFN response is an indirect proof of MC infection, it also witnesses the ability of these cells to effectively clear SARS-CoV-2 infection at later time points. Interestingly, SARS-CoV-2 is sensitive to IFN-β treatment, and it may modulate the onset of the type I IFN response in infected cells through the activity of many viral proteins (Lei et al., 2020).

Differently from *in vitro* studies, autoptic examination of pleura failed to demonstrate infection of MCs by SARS-CoV-2. The almost complete loss of the MC monolayer, the relatively small number of patients examined or the timing of analysis may explain these negative results. To this respect, further study is warranted.

It is known that cytokine production has both a pathogenic and a prognostic role in SARS-CoV-2 infection. Cytokine storm is responsible for multiorgan pathology and eventually death, and inflammatory cytokine signatures may predict COVID-19 severity and patient survival (Del Valle et al., 2020; Mangalmurti and Hunter, 2020).

With respect to cytokine production by MCs, it should be considered these cells are key players in surveying the composition of the fluids covering the serosal membranes and in leukocyte recirculation. In coordination with resident macrophages and other immune cells, MCs secrete inflammatory cytokines and chemokines during bacterial and viral infections due to specific TLR activation, contributing in the shaping of the subsequent immune response (Terri et al., 2021) (Raby et al., 2018).

The analysis of cytokines secreted by SARS-CoV-2-infected MCs highlighted a predominance of anti-inflammatory (i.e., IL-10, sTNF-Rs) over pro-inflammatory (IL-1β, TNFα, IL-6) response. Indeed, while inflammatory cytokine production was negligible (IL-1β, TNFα) or not significantly increased (IL-6), the production of IL-10 and of TNFRs was significantly enhanced upon viral infection. MCs are known to secrete high amounts of IL-6 *in vitro* even in unstimulated conditions, while production of TNF-alpha and IL-1 is more restricted to specific pro-inflammatory stimuli (Topley et al., 1993; Douvdevani et al., 1994; Chen et al., 2015).

Of note, increased expression of sTNF-Rs has been previously reported in septic pleural effusions (Marie et al., 1997). The increase of interferons (IFNα−β−γ and IL-28-29) corresponds to an increase of cytokines with anti-inflammatory/immunomodulatory activity (IL-10, IL-11 and IL-20). During SARS-CoV-2 infection, anti-inflammatory mediators are secreted at the same time with pro-inflammatory mediators, and in particular, IL-10 and IL-6 expression both correlate with disease severity (Han et al., 2020). However, IL-10 appears to have a “double edge” activity during inflammation: this cytokine is a key negative regulator of T cell mediated responses, but is also endowed with pro-inflammatory effects, including stimulation of IFNγ production (Lu et al., 2021).

Moreover, MCs produced significantly increased levels of IL-2 and IL-12 and IL-27, which may both activate NK and Th1 lymphocytes and promote antigen presentation. Interestingly, IL-27 is both an IFNγ-induced cytokine and an activator of IL-10 synthesis (Murugaiyan et al., 2009; Blahoianu et al., 2014).

The production of MMPs by MCs is potentially relevant for induction of the pleura fibrotic response observed during SARS-CoV-2 infection. Finally, another significantly increased cytokine was osteocalcin. This cytokine implicated in ECM remodeling promotes bone formation and may counteract osteoporosis observed in SARS-CoV-2 patients. A loop with IL-6, abundantly expressed by MCs, promotes osteocalcin expression (Chowdhury et al., 2020).

It is conceivable that due to the considerable extension of pleura surface (2000 cm^2^ in an average adult male), the serous fluid recycling and the high vascularization of this organ, the predominant production of cytokines with anti-inflammatory activity by MCs may have a systemic effect of homeostatic dampening of the inflammatory response during infection. On the other hand, pleura may contribute to macrophage/lymphocyte activation and, through the secretion of mediators of ECM remodeling such as MMPs, play a role in lung fibrosis.

Of note, biologic drugs interfering the activity of some of the same cytokines here demonstrated to be produced by infected MCs are being currently analyzed for their therapeutic potential to dampen the noxious inflammatory response (Mangalmurti and Hunter, 2020).

**FIGURE 5:**
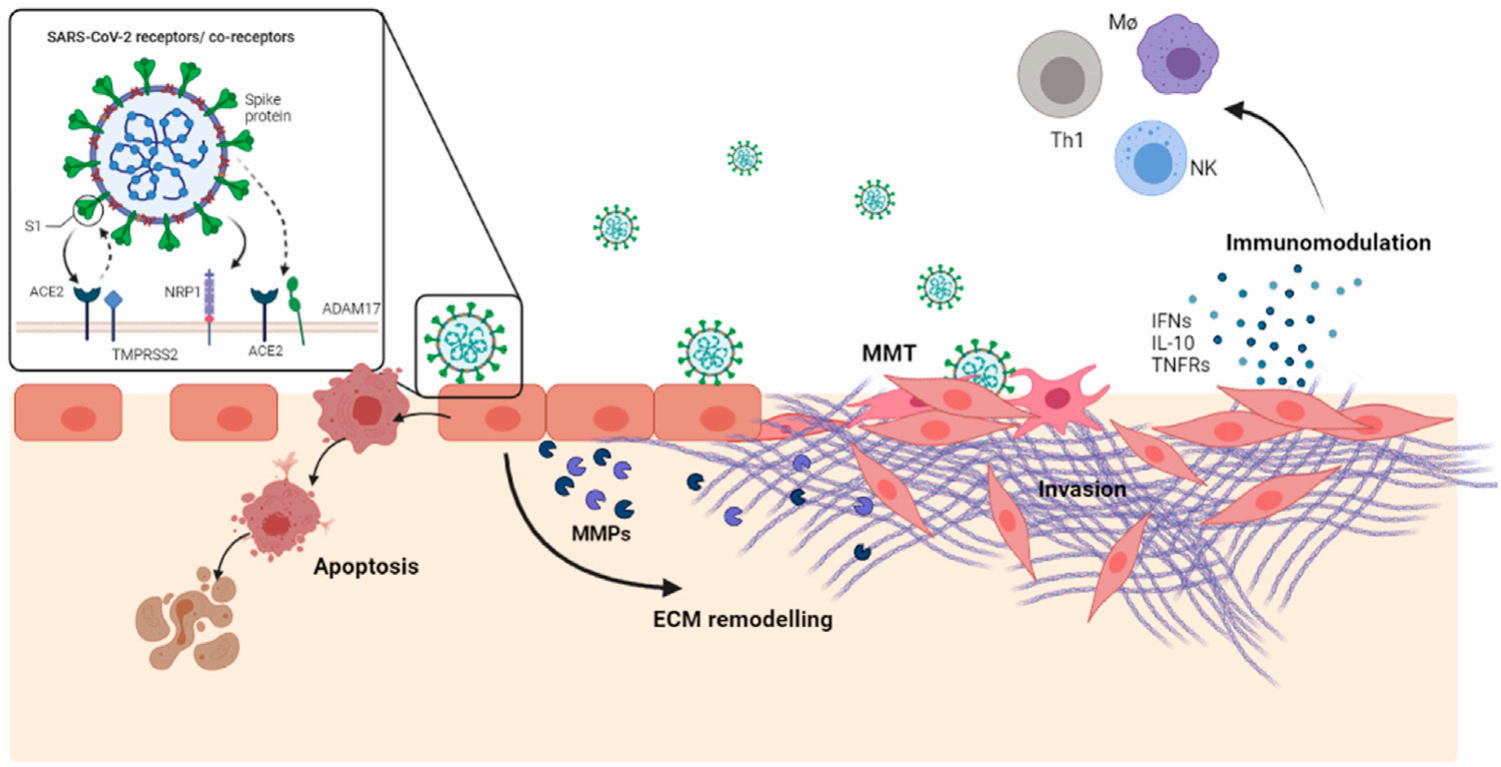
SARS-CoV-2, in combination with other local signals released by the stroma/immune cells, may promote a transient MC infection followed by cell death as well as by induction of MMT. Moreover, infected MCs secrete a number of extracellular mediators active in inflammation, fibrosis immunomodulation.

Overall, our results suggest that a transient MCs infection, in combination with other local signals released by stromal/immune cells, may promote both cell death with pleura disruption, and induction of MMT with the acquisition of an invasive abilities. At the same time, SARS-CoV-2 infection promotes the secretion of cytokines and other extracellular mediators which may both modulate the inflammatory response and induce ECM remodeling. A comprehensive experimental model of MCs response to SARS-CoV-2 infection is shown in Figure 5.

Thus, MCs are candidates for cellular interventions aimed at restoring the continuity of the monolayer, at modulating the anti-viral immune response and at limiting the insurgence of fibrosis. These discoveries warrant more study to better define the role of MCs and their potential implication for therapy.

## Data Availability

The original contributions presented in the study are included in the article/Supplementary Material, further inquiries can be directed to the corresponding author.
