# Supplementary figures and images for: Pleural Mesothelial Cells Modulate the Inflammatory/Profibrotic Response During SARS-CoV-2 Infection
Source: Front Mol Biosci. 2021 Nov 26;8:752616. doi: 10.3389/fmolb.2021.752616 (PMC8662383; doi:10.3389/fmolb.2021.752616)

**A**

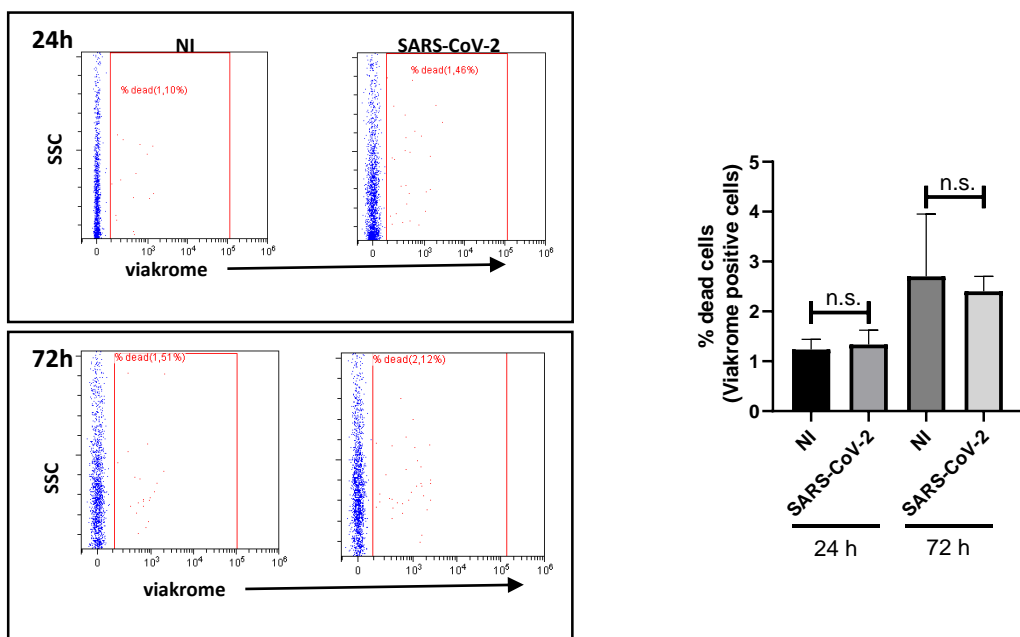

# B

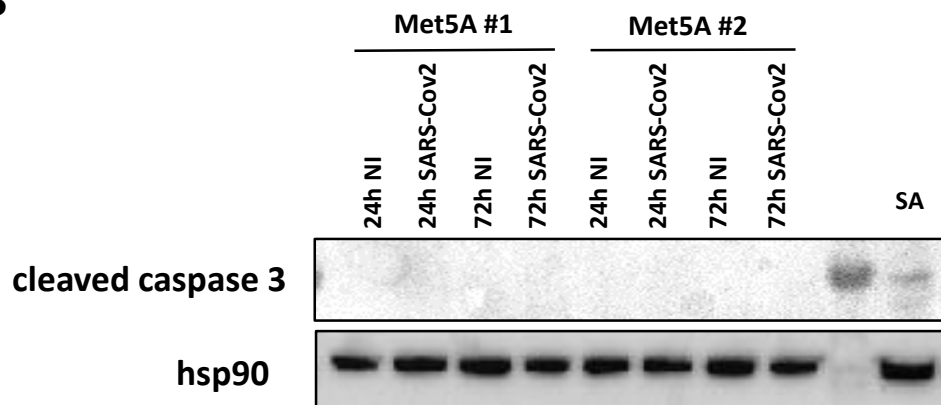

Supplement: Supplementary file 1 [file DataSheet2.PDF]

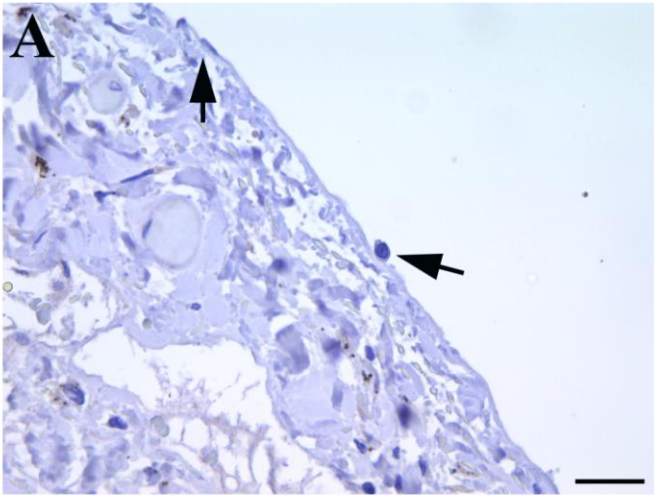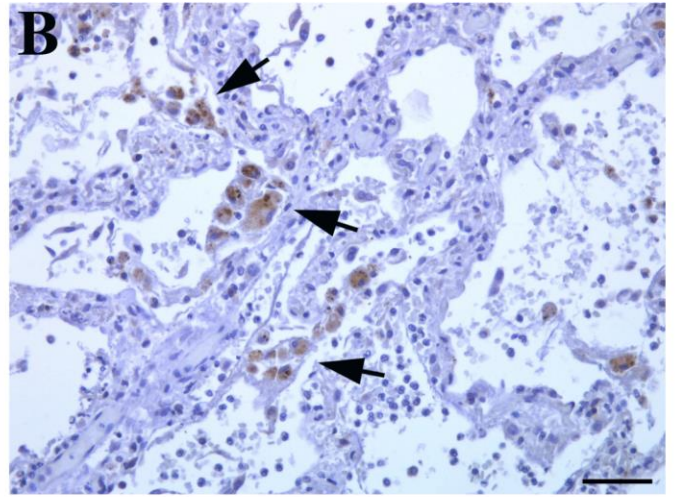

Supplement: Supplementary file 4 [file DataSheet1.PDF]
