# Supplementary material for: Pleural Mesothelial Cells Modulate the Inflammatory/Profibrotic Response During SARS-CoV-2 Infection
Source: Front Mol Biosci. 2021 Nov 26;8:752616. doi: 10.3389/fmolb.2021.752616 (PMC8662383; doi:10.3389/fmolb.2021.752616)
Supplement: Supplementary file 2 [file DataSheet4.PDF]

Supporting information captions

Supplementary figure 1 IHCs showing SARS-CoV-2 detection in lung samples (**A**) IHC anti-SARS-CoV nucleoprotein in COVID-19 autoptic visceral pleura shows no labelling in the epithelial-like MCs (arrows). (**B**) A focal distribution of the positivity is revealed in alveolar lung epithelial cells (arrows). Scale bars: A = 7 μm; B =14 μm.

Supplementary figure 2: (**A)** viability assay of MeT5A cells infected with SARS-CoV-2. Left: Representative Staining at 24-72 h d.p.i using ViaKrome Fixable Viability Dye. Right: quantification of the percentages of positive dead cells in untreated versus SARS-CoV-2 infected MeT5A cells. The experiment was repeated three times. n.s.=not significant (**B)** WB showing the expression of cleaved caspase 3 from total lysates of MeT5A cells infected as in A. Hsp90 was detected as a loading control.SA. Sodium arsenite (2.5 μM) was used as positive control.

Supplementary figure 3: Complete Luminex analysis of supernatant from SARS-Co-V2-infected MeT5A cells
