# Supplementary material for: Pleural Mesothelial Cells Modulate the Inflammatory/Profibrotic Response During SARS-CoV-2 Infection
Source: Front Mol Biosci. 2021 Nov 26;8:752616. doi: 10.3389/fmolb.2021.752616 (PMC8662383; doi:10.3389/fmolb.2021.752616)
Supplement: Supplementary file 3 [file DataSheet3.PDF]

Luminex Assay	MeT5A Experiment 1				MeT5A Experiment 2				MeT5A Experiment 3			
Protein (pg/ml)	24h NI	24h SARS-CoV-2	72h NI	72h SARS-CoV-2	24h NI	24h SARS-CoV-2	72h NI	72h SARS-CoV-2	24h NI	24h SARS-CoV-2	72h NI	72h SARS-CoV-2
**APRIL/TNFSF13**	65940,54	53131,06	47616,11	78167,35	46162,1	54444,46	41576,98	82595,2	58252,52	28942,77	81138,05	80156,39
**BAFF/TNFSF13B**	366,08	124,88	124,88	860,98	124,88	124,88	124,88	854,19	124,88	124,88	1434,24	1707,98
**sCD30/TNFRSF8**	79,84	56,48	25,63	117,91	40,7	46,34	25,11	129,31	52,09	6,7	99,22	116,25
**sCD163**	7089,39	7089,39	7089,39	7089,39	7089,39	7089,39	7089,39	7089,39	7089,39	7089,39	7089,39	7089,39
**Chitinase 3-like 1**	133,58	133,58	133,58	133,58	133,58	133,58	133,58	133,58	133,58	133,58	133,58	133,58
**gp130/sIL-6Rb**	63,84	63,84	63,84	63,84	63,84	63,84	63,84	63,84	63,84	63,84	63,84	63,84
**IFNa2**	112,89	79,5	51,77	150,87	63,12	90,68	51,77	150,87	68,26	24,45	118,92	139,92
**IFNb**	19,59	12,52	6,77	20,24	11,06	13,05	7,18	20,24	12,52	3,04	15,15	18,68
**IFNg**	56,91	46,32	27,03	76,25	33,37	46,97	27,03	84,21	37,22	16,82	56,91	69,06
**IL-2**	65,21	38,93	25,36	81,32	32,99	32,14	25,36	83,02	31,3	14,34	64,36	69,88
**sIL-6Ra**	59,91	59,91	59,91	59,91	59,91	59,91	59,91	59,91	59,91	59,91	59,91	59,91
**IL-8**	639,29	596,33	589,56	752,68	565,67	539,57	607,62	710,29	548,79	560,18	656,72	693,53
**IL-10**	19,25	5,8	5,8	24,71	5,8	5,8	5,8	21,96	5,8	5,8	5,8	25,05
**IL-11**	2889,43	2620,57	375,38	4774,19	2269,8	2574,53	322,23	4730,53	2379,01	245,32	3872,33	4224,99
**IL-12p40**	217,3	157,55	93,9	259,4	126,71	149,45	94,4	264,47	136,81	52,61	207,17	237,58
**IL-12p70**	0,95	0,82	0,49	1,8	0,33	0,82	0,49	1,92	0,7	0,49	1,55	1,43
**IL-19**	227,6	181,42	137,2	281,71	120,97	181,42	23,9	278,82	293,15	23,9	342,63	342,63
**IL-20**	57,81	44,51	31,21	82,07	36,85	41,73	26,94	86,81	42,19	15,76	62,37	74,62
**IL-22**	52,97	46,96	7,11	85,29	32,99	54,94	7,11	83,53	43,86	7,11	62,61	65,42
**IL-26**	885,13	721,03	383,56	1141,47	682,04	701,56	332,42	1122,63	701,56	238,83	971,1	1009,14
**IL-27**	119,36	116,85	88,53	180,2	93,78	109,25	72,46	210,7	104,13	34,81	169,49	187,3
**IL-28A/IFN-L2**	141,8	114,29	67,72	186,7	94,02	100,17	69,47	195,76	140,02	51,98	190,33	239,62
**IL-29/IFN-L1**	271,34	187,44	136,48	319,31	154,47	190,44	121,48	328,31	190,44	85,42	268,35	328,31
**IL-32**	68,99	51,46	34,47	92,06	41,3	51,46	31,78	87,7	49,46	14,43	68,04	77,49
**IL-34**	9459,6	4379,83	2535,67	7148,34	2977,64	1535,77	3963,65	6585,32	2905,88	999,03	6002,42	7030,42
**IL-35**	353,01	305,17	141,52	467,22	254,78	299,53	145,7	500,43	279,3	90,48	388,77	447,17
**LIGHT/TNFSF14**	20,32	16,34	5,36	27,85	12,82	17,67	5,36	31,18	14,14	1,45	20,32	24,97
**MMP-1**	655,46	412,11	181,5	748,64	231,83	381,24	181,5	805,47	251,63	181,5	547,26	667,55
**MMP-2**	5697,02	4657,43	2200,84	8776,09	2200,84	3661,14	1992,8	8776,09	2605,62	801,65	7026,5	8306,55
**MMP-3**	3865,38	3226,72	2344,02	4920,29	2986,93	3265,14	2395,11	5128,36	3340,77	510,01	4151,45	4634,79
**Osteocalcin**	1271,03	1133,56	771,71	1708,29	891,62	1118,23	666,42	1857,47	958,93	463,01	1387,4	1698,32
**Ostepontin (OPN)**	46,19	46,19	46,19	46,19	46,19	46,19	46,19	46,19	46,19	46,19	46,19	46,19
**Pentraxin-3**	96701,45	78800,53	13168,86	163028,88	60155,45	68618,43	14470,84	161542,98	131850,28	13408,42	351168,37	332449,97
**sTNF-R1**	555,33	513,92	751,39	2160,92	563,24	775,85	628,16	2742,41	710,6	225,56	1354,25	1590,32
**sTNF-R2**	92,32	84,38	43,03	120,15	70,82	80,08	45,54	127,55	75,62	21,99	102,98	116,84
**TSLP**	46,16	39,16	22,78	68,16	33,68	38,16	22,28	66,38	34,18	12,45	51,69	56,23
**TWEAK/TNFSF12**	3,08	3,08	3,08	3,08	3,08	3,08	3,08	3,08	3,08	3,08	3,08	3,08
